# The effect of bacteriochlorophyll derivative WST-D and near infrared light on the molecular and fibrillar architecture of the corneal stroma

**DOI:** 10.1038/s41598-020-66869-y

**Published:** 2020-06-17

**Authors:** S. Hayes, N. Aldahlawi, A. L. Marcovich, J. Brekelmans, A. Goz, A. Scherz, R. D. Young, J. S. Bell, D. P. O’Brart, R. M. M. A. Nuijts, K. M. Meek

**Affiliations:** 1 Structural Biophysics Research Group, School of Optometry and Vision Sciences, Cardiff University, Cardiff, United Kingdom; 2 Department of Plant and Environmental Sciences, The Weizmann Institute of Science, Rehovot, Israel; 3 Department of Ophthalmology, Kaplan Medical Center, Rehovot, Israel; 4 University Eye Clinic Maastricht, Maastricht University Medical Center, Maastricht, the Netherlands; 5 Keratoconus Research Institute, Department of Ophthalmology, St Thomas Hospital, London, United Kingdom

**Keywords:** Biophysics, Structural biology, Medical research

## Abstract

A cross-linking technique involving application of Bacteriochlorophyll Derivative WST-11 mixed with dextran (WST-D) to the epithelium-debrided cornea and illumination with Near Infrared (NIR), has been identified as a promising therapy for stiffening pathologically weakened corneas. To investigate its effect on corneal collagen architecture, x-ray scattering and electron microscopy data were collected from paired WST-D/NIR treated and untreated rabbit corneas. The treated eye received 2.5 mg/mL WST-D and was illuminated by a NIR diode laser (755 nm, 10 mW/cm^2^). An increase in corneal thickness (caused by corneal oedema) occurred at 1-day post-treatment but resolved in the majority of cases within 4 days. The epithelium was fully healed after 6–8 days. X-ray scattering revealed no difference in average collagen interfibrillar spacing, fibril diameter, D-periodicity or intermolecular spacing between treated and untreated specimens. Similarly, electron microscopy images of the anterior and posterior stroma in healed WST-D/NIR corneas and untreated controls revealed no obvious differences in collagen organisation or fibril diameter. As the size and organisation of stromal collagen is closely associated with the optical properties of the cornea, the absence of any large-scale changes following treatment confirms the potential of WST-D/NIR therapy as a means of safely stiffening the cornea.

## Introduction

The healthy cornea, by virtue of its stiffness, transparency and precise curvature, provides the eye with the majority of its focussing power and facilitates the transmission of light onto the retina. These intrinsic properties are largely governed by the specific arrangement of collagen within its stroma - a layer which occupies approximately 90% of the entire thickness of the tissue. Within the stroma, long, thin collagen fibrils are arranged parallel to each other in layers (lamellae), which are themselves stacked parallel to the corneal surface in the posterior stroma but become increasingly interwoven with proximity to the anterior surface of the cornea^1^. Transparency is largely dependent on the narrow diameter and short-range order of the constituent collagen fibrils^2^, both of which are thought to be regulated by close interactions with proteoglycans^3,4^. The flat cells (keratocytes) that exist between the collagen lamellae, also play a role in tissue transparency, as the refractive index of their cytoplasm closely matches that of the stroma as a whole^5^. Activation of the normally quiescent keratocytes during wound healing results in a mismatch between the refractive indices of the keratocytes and the extracellular matrix that is believed to contribute to an observed increase in light scatter^5,6^.

In the condition keratoconus, the corneal stroma becomes progressively thin and weak and deforms outwards. This leads to a steepening of the cornea and severe, irregular astigmatism^7^. The precise cause of keratoconus and the mechanism by which it progresses remain uncertain but it is thought to involve both enzymatic digestion and lamellar slippage^8,9^. Over the past decade, riboflavin/UVA cross-linking therapy has been shown to effectively halt keratoconus progression^10^ by increasing the stiffness^11^ and enzymatic resistance^12,13^ of the cornea. However, the treatment, which involves debridement of the epithelium followed by stromal absorption of riboflavin and irradiation with UVA, is not recommended for corneas with a thickness of less than 400 µm due to the risk of endothelial cell damage^14,15^. Furthermore, there have been rare reports of side-effects such as corneal haze, keratitis and corneal scarring occurring after treatment^16^.

With the aim of producing a treatment capable of safely stiffening diseased or surgically weakened corneas of all thicknesses, researchers at the Weizmann Institute of Science investigated the potential of synthesized chemical derivatives of photosynthetic pigments (chlorophylls and bacteriochlorophylls). It was found that a 20 minute topical application of palladium bacteriochlorin 13′-(2-sulfoethyl)amide dipotassium salt (WST11)^17^ in 20% dextran T-500 (WST-D) followed by a 30 minute illumination at 755 nm (NIR) resulted in an increase in rabbit corneal stiffness that was comparable in magnitude to that achieved with the standard riboflavin/UVA protocol^18^. Moreover, examination of WST-D/NIR treated rabbit corneas at 1, 4 and 8-months follow-up confirmed the stiffening effect to be persistent and long-term^19^.

On the basis that corneal function is largely dependent on the structural organisation of the stromal extracellular matrix, this study used both synchrotron x-ray scattering and transmission electron microscopy to assess the overall impact of WST-D/NIR therapy on collagen fibril architecture as an average throughout the whole tissue thickness, and at a highly localised level. In doing so, we aimed to provide insight into the means by which WST-D/NIR therapy stiffens the cornea and assess its potential as a clinical treatment.

## Methods

Table 1 provides a summary of the samples, treatments and data collection methods described below.

**Table 1:** Specimen treatments and data collection.

Rabbit	Treatment	Data collection method (n = number of paired treated/untreated corneas examined)
1–12	*Ex vivo*: Untreated WST-D/NIR	Small-angle x-ray scattering (n = 12)
13–16	*In vivo*: Untreated WST-D/NIR	Wide-angle x-ray scattering (n = 4)
17–22	*In vivo*: Untreated WST-D/NIR	Small-angle x-ray scattering (n = 6)
23–24	*In vivo*: Untreated WST-D/NIR	Electron microscopy (n = 2)
25–26	*In vivo*: Untreated Riboflavin/UVA	Electron microscopy (n = 2)

### *Ex vivo* tissue for x-ray scattering studies (samples 1–12)

Twelve pairs of rabbit eyes were obtained within 2 hours of death from an abattoir and treated *ex vivo*. The right eye of each pair served as an untreated control, whilst the left eye underwent corneal de-epithelialisation and WST-D/NIR cross-linking. The cross-linking procedure involved a 20-minute impregnation with WST-D (containing 20% dextran T-500) using a 12 mm diameter eye cap, followed by NIR illumination (755 nm, 10 mW/cm^2^) for 30 minutes. After treatment, the cornea (with a 2 mm scleral rim) was trephined from each eye, wrapped in polyvinyl chloride catering film to prevent moisture loss, and transferred to −80 °C storage. The specimens remained frozen during transportation to the Diamond Light Source synchrotron (Didcot, UK) and were maintained in this state until required for small-angle x-ray scattering data collection.

### *In vivo* tissue for x-ray scattering studies (samples 13–22)

In accordance with the Association for Research in Vision and Ophthalmology Statement on the Use of Animals in Ophthalmic and Vision Research and following approval from the Institutional Animal Care and Use Committee at the Weizmann Institute of Science, 10 New Zealand White rabbits (15 weeks old, 2.7–2.9 kg) were anesthetized by intramuscular injection of 35 mg/kg ketamine (Rhone Merieux, Lyon, France) and 5 mg/kg xylazine (Vitamed, Benyamina, Israel). The right eye of each animal served as an untreated control, whilst the left eye underwent removal of the corneal epithelium and WST-D/NIR cross-linking, in the manner described previously for *ex vivo* corneas. An aluminium foil mask with an 8 mm diameter central opening was used to restrict the illuminated area and prevent exposure of the limbal stem cells. In 4 out of 10 rabbits, pachymetry was performed before treatment, immediately after treatment, and at day 1, 5, 10, and 20 post-treatment using an ultrasonic pachymeter (Humphrey ultrasonic pachymeter, USA). In the remaining 6 rabbits, pachymetry was carried out before treatment and at 28 days post treatment. An ophthalmic steroid and antibiotic ointment containing dexamethasone 0.1%, neomycine and polymixin B (Maxitrol, Alcon, Belgium) was applied once daily to each treated eye for a period of 7–10 days until complete re-epithelialisation had occurred. At 28 days post-treatment the rabbits were euthanized. The corneo-scleral discs (cornea with a 2-mm scleral rim) were removed, placed in storage medium and transported to Diamond Light Source for examination on small- and wide-angle x-ray scattering beamlines (I22 and I02 respectively). Due to beamline access restrictions, the 4 pairs of corneo-scleral discs destined for wide-angle x-ray scattering studies (samples 13–16) were stored in Optisol (Chiron Ophthalmics, Irvine, California) for 8 days prior to data collection and the 6 pairs of corneo-scleral discs for small-angle x-ray scattering studies (samples 17–22) were stored in minimal essential medium with 6% dextran for 2 days prior to data collection.

### Small-angle x-ray scattering data collection and analysis (samples 1–12 and 17–22)

**Figure 1:**
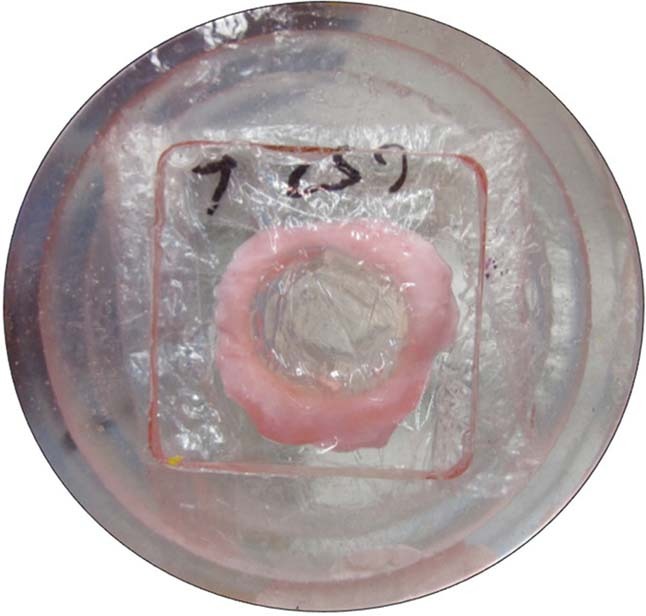
An untreated rabbit cornea (with scleral rim), wrapped in catering film and enclosed within a custom-made Perspex sample holder with two Mylar (Dupont-Teijin, UK) windows. This design ensures minimal tissue dehydration during x-ray data collection.

Thirty minutes prior to x-ray data collection, the 12 pairs of frozen *ex vivo* prepared corneo-scleral discs were thawed at room temperature, after which a 6-mm disc was trephined from the centre of each cornea. Similarly, a 6-mm disc was also trephined from the centre of each of 6 pairs of *in vivo* prepared corneas. The corneal discs were weighed, re-wrapped in catering film (to prevent tissue dehydration) and enclosed within a custom-made sample holder (Fig. 1). Small-angle x-ray scattering data, obtained from the centre of each corneal disc using a 0.5 s exposure to a 180 ×300 µm x-ray beam (λ = 0.1 nm), were recorded on a detector positioned 5.8 m behind the sample.

Small-angle x-ray scattering patterns were calibrated against the 58.380 Å d_001_ lattice reflection in powder diffraction patterns of silver behenate. Using methods detailed previously, data were analysed using an integrated software tool specifically designed for the analysis of fibrous collagen-based tissues (SAXS4COLL), to generate average measurements of collagen D-periodicity, fibril diameter and inter-fibrillar spacing^20^.

As collagen inter-fibrillar spacing is particularly sensitive to changes in corneal hydration^21^, it is essential to discriminate between hydration and treatment induced changes in corneal ultrastructure. In order to determine the extent of tissue drying during the data collection process, wet mass measurements for 8 of the *ex vivo* prepared corneal discs were recorded immediately before and after x-ray exposure. Following data collection, all of the 6-mm corneal discs were oven dried at 60 °C for 7 days and re-weighed. Measurements of wet and dry mass were used to calculate the hydration (H) of the tissue at the time of data collection using Eq. (1):

1 $$H=\frac{{m}_{w}-{m}_{d}}{{m}_{d}}$$

where ${m}_{w}$ and ${m}_{d}$ are the wet and dry masses of the corneal disc, respectively.

### Wide-angle x-ray scattering data collection and analysis (samples 13–16)

Four pairs of *in vivo* treated/untreated corneo-scleral discs were removed from their storage solution, wrapped in catering film and placed within a custom-made Perspex sample holder with mylar windows. Wide-angle x-ray scattering patterns, resulting from a 0.5 s exposure to a 52 ×83 µm x-ray beam with a wavelength of 0.1 nm, were obtained at 1 mm intervals over the central 5 mm of each corneo-scleral disc, and recorded on a detector positioned 350 mm behind the sample. Following data collection, a 6 mm biopsy was trephined from the centre of each cornea and its wet mass measured. A dry mass measurement of the biopsy was obtained following storage at 60 °C for 7 days, allowing the hydration of the specimen at the time of data collection to be calculated using Eq. (1).

Using the analysis technique described previously^20^, wide-angle x-ray scatter patterns were calibrated against the 0.305 nm d_104_ lattice reflection in powder diffraction patterns of calcium carbonate and the modal average inter-molecular spacing of corneal collagen was determined from the position of the intermolecular equatorial reflection peak. The relationship between x-ray Bragg spacing and the corresponding centre-to-centre distance of the parameter under investigation (collagen inter-molecular or inter-fibrillar spacing) depends on the precise packing of the molecules within the fibrils, or of the fibrils within the stroma. Most previous investigations have assumed a liquid-like packing^22,23^, in which case the Bragg spacing needs to be multiplied by a factor of 1.1–1.2 in order to convert to centre-to-centre spacing. However, as we are only concerned here with fractional changes in these parameters, all small and wide-angle x-ray scattering results are presented as Bragg spacing.

### *In vivo* tissue for electron microscopy studies (samples 23–26)

Four, 20-week old (3–3.5 Kg), female, New Zealand White rabbits were anesthetized as described previously. In each case the right eye remained as an untreated control, whilst the corneal epithelium was removed from the left eye. In two of the rabbits (sample 23–24), WST-D/NIR cross-linking (as described previously for *ex vivo* corneas) was performed on the de-epithelialized corneas. In the remaining two rabbits (sample 25–26), the de-epithelialized corneas underwent riboflavin/UVA cross-linking which involved a 30-minute pre-treatment with a commercial riboflavin solution (containing 0.1% riboflavin and 20% dextran (Mediocross, Germany), followed by a 30 minute UVA illumination with a 365 nm diode at 3 mW/cm^2^. All WST-D/NIR and riboflavin/UVA cross-linked eyes were treated with Maxitrol (steroid and antibiotic) ointment daily for 2-weeks until the corneal epithelium had fully recovered. Measurements of corneal thickness, made using an ultrasound pachymeter, were recorded before treatment and immediately prior to euthanasia, which occurred at one month. Following sacrifice, the corneas were removed and processed for electron microscopy as described below.

### Electron microscopy data collection and analysis

Corneas were fixed in 2.5% glutaraldehyde in 0.1 M Sorensen phosphate buffer (pH 7.2–7.4) for 3 hours on a rotator then washed twice and stored in buffer at 4 °C overnight. The corneal tissue was then prepared for transmission electron microscopy. The tissue was post fixed in 1% osmium tetroxide in 0.1 M Sorensen buffer for 1 hour, washed in distilled water, placed in 1% aqueous uranyl acetate for 1 hour and then re-washed with distilled water. Specimens were subjected to ethanol series dehydration (from 70%, through 90% to 100%) and, via propylene oxide, infiltrated and embedded at 60 °C in epoxy resin over two days (Araldite CY212 resin, TAAB Laboratories, England, UK). A Leica UC6 ultramicrotome was used to cut ultrathin sections (90–100 nm thick), which were stained with uranyl acetate and lead citrate for examination in a JEM 1010 transmission electron microscope (Jeol (UK) Ltd, Welwyn Garden City, UK). Transmission electron microscopy images were collected from the anterior and posterior regions of each cornea and then analysed in Image J^24,25^. Using this software, circles were manually fitted to 105 individually distinguishable fibrils in both the anterior and posterior stroma of each cornea, allowing the average diameter of fibrils to be calculated for each region. Only fibrils cut orthogonally were included in the analysis, those cut at oblique angles were omitted from the study.

### Statistical analysis

Statistical differences between paired samples (treated and untreated fellow eyes) and between measurements recorded from the same samples at multiple time points (e.g. pachymetry measurements) were assessed by means of paired student t-tests. In cases where paired data were unavailable, statistical differences were assessed by means of a non-paired student t-test. Statistical analyses between different treatment groups were performed using a one-way ANOVA followed by Sidak *post hoc* test and/or Bonferroni *post hoc* test. All statistical analyses were performed using a Statistical Package for the Social Sciences (SPSS Statistics 20; IBM, Armonk, NY, USA). A probability value of p < 0.05 was considered significant. Values presented are means ± standard deviation (SD).

## Results

### Clinical observations and corneal thickness measurements

**Figure 2:**
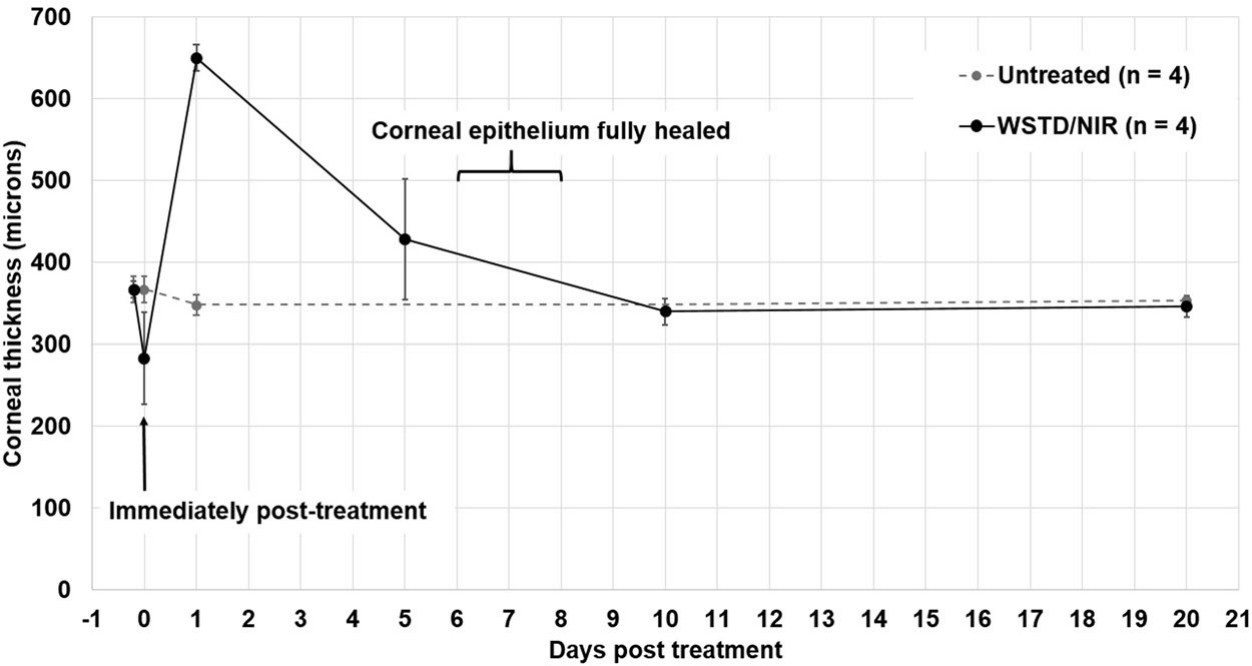
Average corneal thickness immediately pre- and post- WST-D/NIR treatment and during healing.

**Figure 3:**
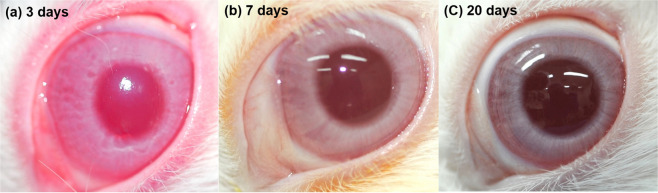
Photographic images of a WST-D/NIR treated cornea at 3, 7 and 20 days’ post-surgery. The 5 mm epithelial erosion which was evident 3 days after surgery (A) was fully healed within one week (B). After 20 days the cornea was transparent (C).

In the *ex vivo* treated corneas (samples 1–12) a significant decrease in corneal thickness was recorded following epithelial debridement and WST-D/NIR treatment (p < 0.0001) (Table 2). Similarly, in the 4 *in vivo* treated rabbits from which pachymetry readings were recorded before and immediately after treatment (samples 13–16), corneal thickness was seen to decrease in all specimens following treatment (p < 0.05) (Table 1 and Fig. 2). This was followed by a significant increase in corneal thickness, as a result of corneal oedema at one-day post treatment (p < 0.001) (Fig. 2). In three out of four of the rabbits, the oedema resolved within 4 days of treatment (Fig. 3A) but in one rabbit, moderate corneal oedema persisted for 9 days. The average corneal thickness of the four treated corneas measured at 5 days post-treatment did not differ significantly from pre-treatment values (Fig. 2). Although a green colouration of the cornea and a slight reduction in tissue transparency was evident immediately after treatment, both issues resolved within 1–2 days and 4–7 days respectively in all corneas (Fig. 3A–C). In all *in vivo* WST-D/NIR treated rabbits (n = 14), the corneal epithelium was fully healed within 6–8 days of treatment (Fig. 3B). At the time of enucleation (20–28 days post-treatment), the thickness of each cornea did not differ significantly from that of its pre-treatment value (Table 2).

**Table 2:** Thickness measurements of *in vivo* and *ex vivo* treated and untreated corneas.

	Pre-treatment (µm)	Immediately post-treatment (µm)	Day 20 post-treatment (µm)	Day 28 post-treatment (µm)
***Ex vivo*: small angle x-ray scattering studies (samples 1–12)**				
Untreated (n = 12)	383 ± 18	383 ± 18	—	—
WST-D/NIR (n = 12)	386 ± 23	364 ± 13	—	—
***In vivo*: small and wide-angle x-ray scattering studies (samples 13–22)**				
Untreated (n = 4)	367 ± 16	367 ± 16	353 ± 4	—
WST-D/NIR (n = 4)	367 ± 8	283 ± 56	346 ± 16	—
Untreated (n = 6)	368 ± 34	—	386 ± 30	—
WST-D/NIR (n = 6)	368 ± 34	—	370 ± 20	—
***In vivo*: electron microscopy studies (samples 23–26)**				
Untreated (n = 2)	383–384	—	—	386–387
WST-D/NIR (n = 2)	380–383	—	—	361, 377
Untreated (n = 2)	398, 384	—	—	375, 386
Ribo/UVA (n = 2)	381, 380	—	—	409, 377

### Collagen intermolecular spacing

Unfortunately, due to technical problems during data collection, the wide-angle x-ray scattering data from one untreated cornea (14 R) and one WST-D/NIR treated cornea (15 L) were unsuitable for analysis. However, analysis of the x-ray scatter patterns from the central 5 mm of the remaining corneas revealed no significant difference in the average collagen intermolecular spacing of WST-D/NIR treated and untreated corneas (Table 3).

**Table 3:** Average collagen intermolecular spacing (IMS) in the central 5 mm region of WST-D/NIR treated and untreated corneas.

Rabbit	Untreated right eye (R)		WST-D/NIR treated left eye (L)	
	Hydration	IMS (nm)	Hydration	IMS (nm)
13	2.7	1.58 ± 0.01	2.7	1.58 ± 0.010
14	—	—	3.1	1.60 ± 0.008
15	3.3	1.60 ± 0.01	—	—
16	2.7	1.57 ± 0.01	2.8	1.60 ± 0.007

### Collagen interfibrillar spacing, fibril diameter and D-periodicity

The average hydration of the *in vivo* samples in the small-angle x-ray scattering study (Table 3) was significantly lower than that of the *in vivo* samples in the wide-angle-x-ray scattering (Table 4) due to differences in sample storage media and duration of storage prior to data collection. Within the *in vivo* and *ex vivo* groups of the small-angle x-ray scattering study component, the hydration of the paired WST-D/NIR treated and untreated corneas did not differ significantly from each other when measured immediately before x-ray scattering data collection (Table 4). Furthermore, wet mass measurements recorded from *ex vivo* corneal buttons before and after the collection of small-angle x-ray scattering data revealed no significant change in corneal hydration during data collection (data not shown).

**Table 4:** Average collagen interfibrillar spacing (IFS), fibril diameter and D-periodicity in WST-D/NIR treated and untreated corneas.

	*Ex vivo*		*In vivo*	
	Untreated (n = 12)	WST-D/NIR (n = 12)	Untreated (n = 6)	WST-D/NIR (n = 6)
Hydration	4.5 ± 0.5	4.6 ± 0.5	4.1 ± 0.4	4.2 ± 0.3
IFS (nm)	61.7 ± 2.6	62.3 ± 2.8	56.4 ± 0.8	55.3 ± 1.2
Fibril diameter (nm)	39.3 ± 0.6	39.4 ± 0.6	42.0 ± 1.1	41.8 ± 1.5
D-period (nm)	65.9 ± 0.7	65.9 ± 0.7	65.9 ± 0.7	65.9 ± 0.7

Using calibrated small-angle x-ray scatter patterns from each WST-D/NIR treated and untreated cornea, the average interfibrillar spacing, fibril diameter and D-periodicity of the collagen fibrils were calculated (Table 4). For each of these collagen parameters there was no significant difference detected between the WST-D/NIR treated corneas and their fellow untreated corneas.

### Electron microscopy

**Figure 4:**
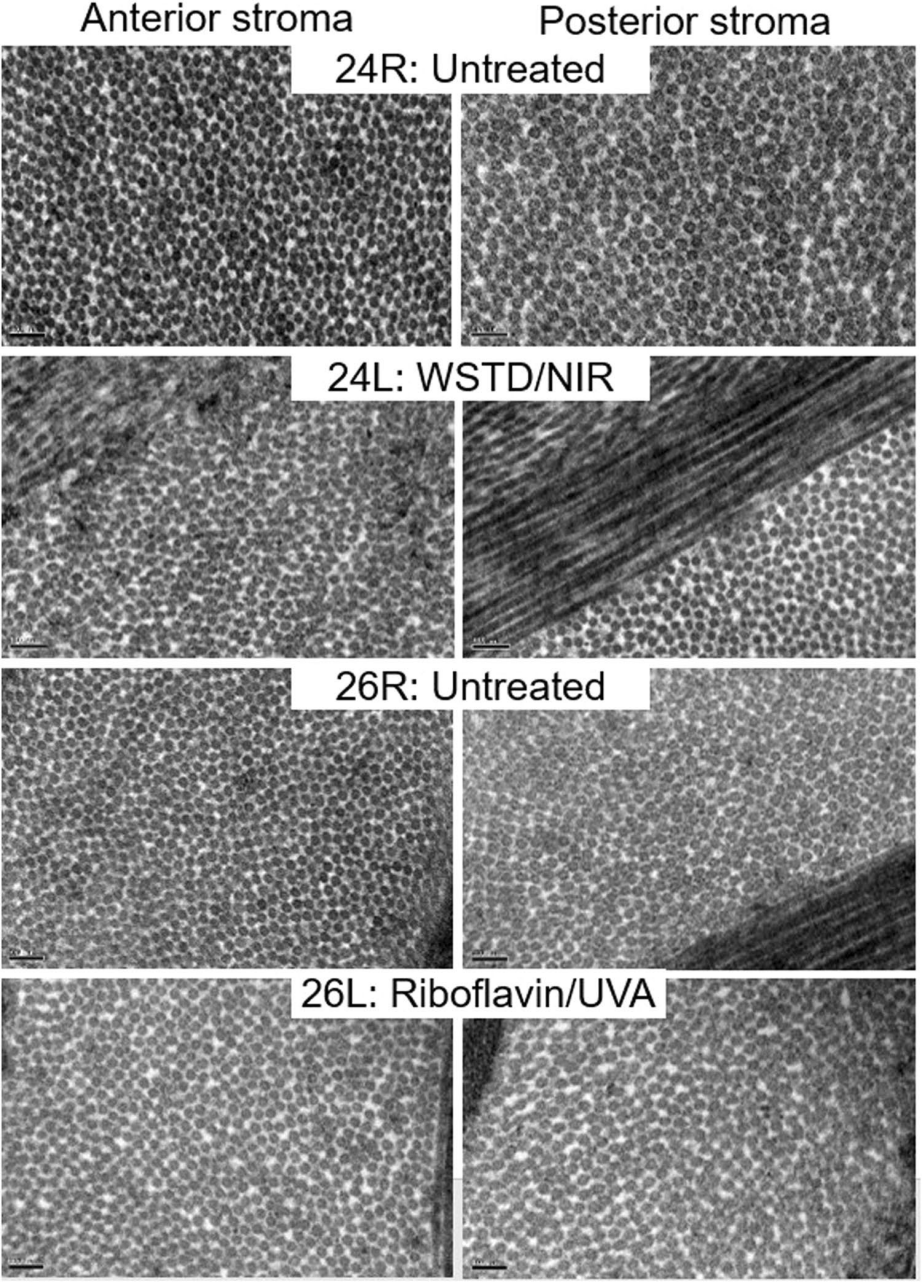
Transmission electron microscopy images obtained from the anterior and posterior stroma of a riboflavin/UVA treated and untreated cornea and a WST-D/NIR treated and untreated cornea. Scale bar = 100 nm.

**Figure 5:**
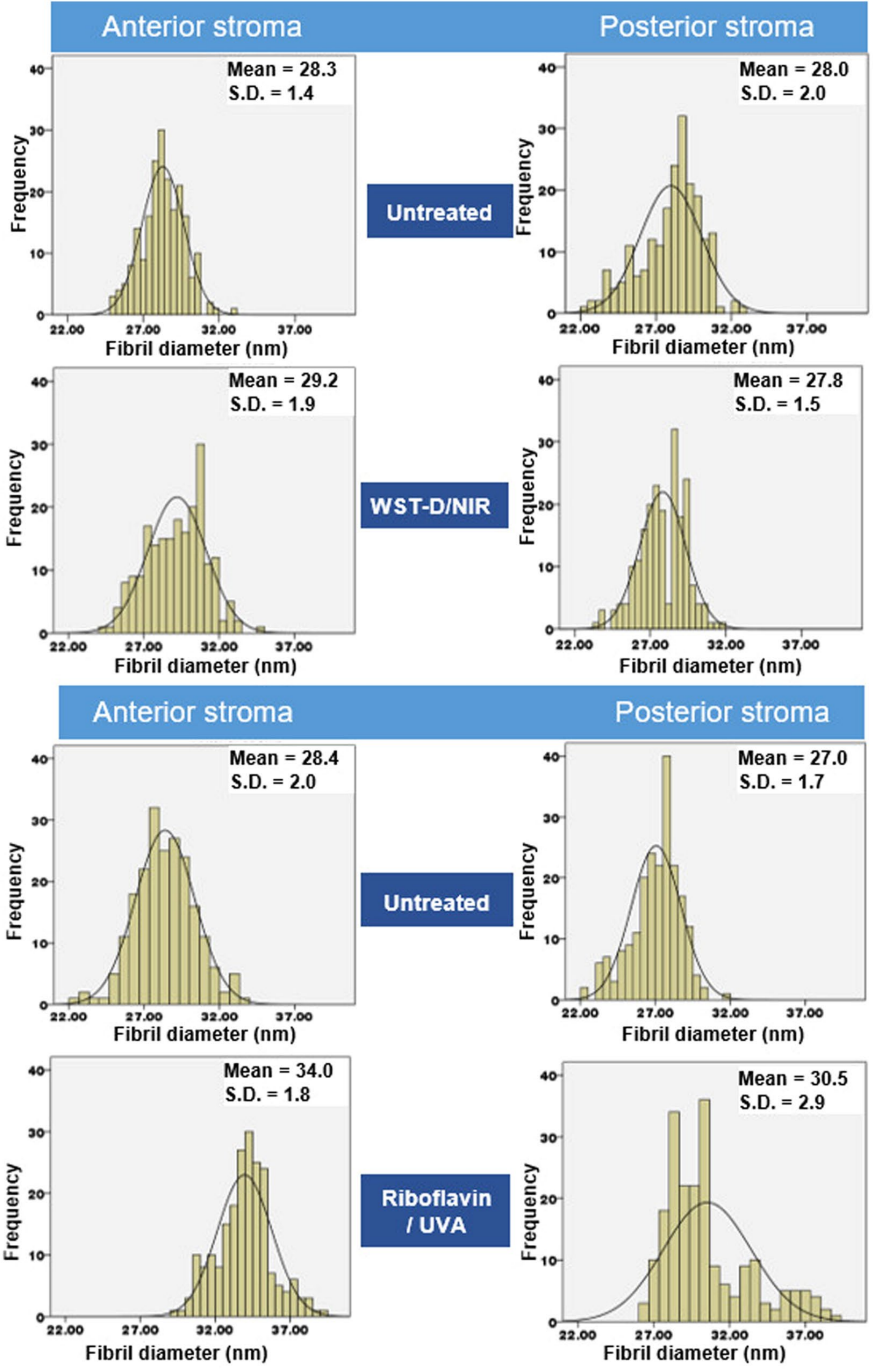
Histograms based on fibril diameter measurements obtained from transmission electron microscopy images of the anterior and posterior stroma in riboflavin/UVA and WST-D treated corneas and contralateral untreated corneas.

Electron microscopy images obtained from the anterior and posterior stromal regions of two pairs of WST-D/NIR treated and untreated corneas and two pairs of riboflavin/UVA treated and untreated corneas revealed no obvious differences in their stromal collagen packing arrangement as a function of tissue depth, or as result of their respective treatments (Fig. 4). However, measurements of fibril diameter obtained from the electron microscopy images showed regional differences within each cornea, with the average diameter of the fibrils being consistently larger in the anterior stroma than the posterior stroma of both the treated and untreated corneas (Fig. 5).

A negligible difference in fibril diameter (<2%) was detected between the WST-D/NIR treated corneas and their untreated controls (Fig. 5), but in the riboflavin/UVA treated corneas the fibrils were found to be on average 16% larger in the anterior stroma, and 12% larger in the posterior stroma, than those present in contralateral, untreated corneas.

**Figure 6:**
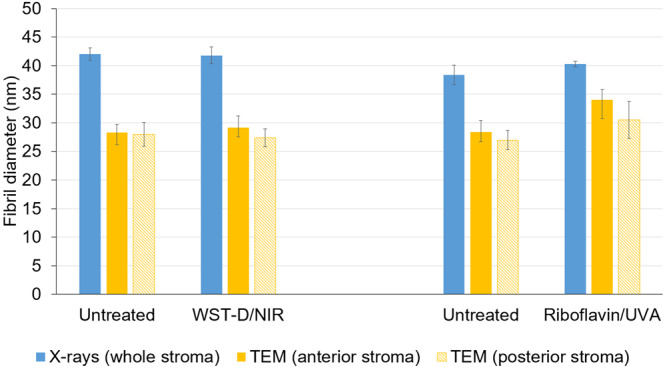
Comparison of fibril diameter measurements obtained from x-ray scattering patterns (averaged throughout the whole stromal thickness) and transmission electron microscopy (TEM) images of the anterior and posterior stroma in WST-D/NIR, riboflavin/UVA-treated and contralateral, untreated corneas. X-ray scattering measurements of riboflavin/UVA treated and untreated rabbit corneas are from previously published data^26^.

Interestingly, electron microscopy derived measurements of fibril diameter in the WST-D/NIR treated corneas and the untreated corneas were approximately 30% smaller than those obtained by x-ray scattering (Fig. 6). However, in riboflavin/UVA treated corneas, the electron microscopy measurements of fibril diameter were only 20% lower than our previously documented x-ray scattering measurements ^27^ from similarly treated (and hydrated) rabbit corneas (Fig. 6).

## Discussion

Akin to riboflavin/UVA cross-linking, WST-D/NIR has been shown to significantly enhance corneal stiffness^18,19^. However, the precise way in which these therapies alter the biomechanical properties of the cornea is not yet fully understood. In an earlier study we provided evidence of enhanced enzymatic resistance in riboflavin/UVA cross-linked corneas but found no change in the swelling behaviour of the stroma, or in the size and organisation of the constituent collagen fibrils when measured by x-ray scattering^26^. These findings, along with those from other studies^27^, led us to believe that the riboflavin/UVA stiffening effect is due to the formation of cross-links at the surface of the fibrils and within the proteoglycan-rich coating surrounding them^26^. Here, we employed some of the same experimental techniques to examine the effect of WST-D/NIR cross-linking on corneal structure and in particular, collagen fibril architecture, in order to gain further insight into its potential as a clinical treatment and the means by which it stiffens the cornea.

In accordance with our previous studies of WST-D/NIR therapy^18^, significant corneal oedema was observed 1 day after treatment but in most cases resolved within 4 days. The cause of this initial swelling cannot be attributed to changes in the endothelial transport properties of the cornea, as our earlier work has shown that the endothelium is unaffected by the WST-D/NIR therapy^18^. Instead, the swelling is most likely caused by the removal of the epithelium, with loss of its barrier function leading to the diffusion of tears into the exposed stroma. Similar changes in corneal hydration to those observed in the present study have been reported by others following the removal and subsequent healing of the rabbit corneal epithelium^28^. Further to this, it has been shown that epithelial debridement causes apoptosis of anterior stromal keratocytes^29^ and it has been suggested that hyperosmotic products of proteolysis may be released from these keratocytes as they undergo apoptosis^30,31^. Such a mechanism may also contribute to the oedema in epithelium-debrided, WST-D/NIR treated corneas, since significant keratocyte apoptosis has been seen histologically in the anterior stroma of these corneas at 2 days post treatment^18^. In all *in vivo* WST-D/NIR treated rabbit corneas, the thickness of the tissue returned to pre-treatment values by day 10 and at the time of x-ray data collection, their hydration was equal to that of their fellow untreated corneas. Since the *ex vivo* WST-D/NIR treated corneas were also found to have a similar hydration to the *ex vivo* untreated corneas, this suggests that the initial decrease in corneal thickness observed immediately post-treatment can be attributed entirely to removal of the epithelium and not to stromal dehydration by the dextran within the WST-D solution.

As demonstrated here and elsewhere^32,33^, electron microscopical examination of untreated rabbit corneas revealed a tendency for the average diameter of collagen fibrils to be slightly larger in the anterior stroma than the posterior. Although this feature is not common to the human cornea^34,35^ it has been observed in other species, such as the cow^36^. Collagen fibril diameter measurements are known to vary significantly between species^37^, but the precise values reported are also dependent on the measurement technique used and the level of tissue processing required^38^. For instance, our electron microscopy-derived measurements of collagen fibril diameter in untreated rabbit corneas were ~30% smaller than those obtained by x-ray scattering, a difference which may be explained by the hydration of the tissue at the time of examination. X-ray scattering measurements are made at close to physiological hydration, whereas, electron microscopy measurements are performed on processed tissue that has been dehydrated to below the critical point of hydration (H < 1.5), when water is lost from both the inter-fibrillar and intra-fibrillar spaces^38,39^. Using x-ray scattering data collected from human and pig corneas during the process of tissue dehydration, we have previously demonstrated that a decrease in collagen fibril diameter occurs prior to any reduction in intermolecular spacing^21^. On this basis, we proposed that x-ray scattering measurements of fibril diameter include the collagen fibril core plus a contribution from the proteoglycan-rich coating surrounding it, the thickness of which is hydration and species dependent^21^. If this hypothesis is correct, then the reduction in collagen fibril diameter that occurs as a result of electron microscopy processing must be due to a shrinkage of the outer coating as well as the fibril core. It follows therefore, that the presence of additional cross-links at the surface of, and within the outer coating of the fibril, may restrict the amount by which the fibrils and their coating can shrink during the dehydration stage of electron microscopy tissue processing. This could explain why collagen fibrils in riboflavin/UVA treated corneas have the same diameter as that of their fellow untreated controls when examined with x-ray scattering^26^ but appear to have comparatively larger than normal fibrils when examined in their dehydrated state by electron microscopy^32^. The magnitude of the discrepancy in fibril diameter measurements between the two techniques may therefore provide an indication of the extent of crosslinking within and around the fibrils. In line with this, our current electron microscopy measurements found the average diameter of collagen fibrils in the anterior and posterior stroma of riboflavin/UVA cross-linked rabbit corneas to be only 16% and 24% lower respectively than our previously published x-ray scattering measurements^26^, whereas in untreated corneas the fibrils appeared ~30% smaller in both regions. The appearance of treatment-induced effects in both the anterior and posterior stromal regions of the riboflavin/UVA cross-linked corneas can be explained by the fact that the depth of the cross-linking is thought to be ~300 µm, thereby encompassing ¾ of the total thickness of the rabbit cornea^40^. However, the reduced fibril shrinkage in the anterior stroma of riboflavin/UVA cross-linked corneas (compared to the posterior stroma) is consistent with the gradual reduction in cross-linking with tissue depth, which is caused by the absorption of UVA by riboflavin and the progressive reduction in UVA irradiance with increasing distance from the anterior stromal surface^41^.

As we have shown previously to be the case for rabbit and porcine riboflavin/UVA cross-linked corneas^26^, x-ray scattering studies of WST-D/NIR treated rabbit corneas also showed no treatment-induced changes in any of our measured collagen parameters (inter-molecular spacing, D-periodicity, fibril diameter and spacing). This suggests that neither of the treatments causes a wide-spread cross-linking of collagen molecules or a change in the axial stagger or tilt of the molecules, and at normal levels of hydration, the distribution of water within and between the collagen fibrils is unaffected by either of the treatments. However, in contrast to the riboflavin/UVA treated corneas, electron microscopy measurements of fibril diameter in the WST-D/NIR treated corneas did not differ from those of untreated corneas in either the anterior or posterior stroma. The absence of any treatment effects in the posterior stroma of the WST-D/NIR corneas is not wholly surprising as the treatment depth is thought to be confined to the anterior ~200 µm of the rabbit corneal stroma^18^. However, the absence of any differences in the anterior stroma of the WST-D/NIR treated corneas suggests that the treatment confers little, if any, resistance to fibril shrinkage, and the induced cross-links may differ from those formed during riboflavin/UVA in terms of their location, number and/or ability to resist fibril shrinkage. Whilst care must be taken to avoid over interpretation of these electron microscopy findings from a relatively small number of samples and further confirmatory studies are clearly warranted, it is interesting to note that such variations in the location of the induced cross-links are supported by fluorescence spectroscopy studies, which have shown that the dityrosine bond fluorescence in riboflavin/UVA treated corneas (indicative of a photodynamic modification of tyrosine, an amino acid residue in the collagen molecule), is absent in WST-D/NIR treated corneas^18^.

Although the findings from this study were unable to confirm where cross-links are formed during WST-D/NIR therapy, the absence of any lasting changes in either the thickness/hydration of the cornea and the size and spacing of the collagen fibrils (as well as the molecules within them), suggests that the treatment-induced corneal stiffening is not due to a thickening of the constituent collagen fibrils or an increase in the total number of fibrils. The latter is not wholly surprising based on the immediate stiffening that occurs in *ex vivo* treated corneas and the fact that biostimulation in other tissues, such as healing skin, typically occurs after multiple, daily treatments of much higher radiant exposures (up to 40 J/cm^2^)^42^ than those experienced during WST-D/NIR therapy.

As the presence of uniformly narrow, regularly spaced collagen fibrils is an essential requirement for corneal transparency, the absence of any large-scale changes in these parameters following WST-D/NIR treatment highlights its potential as a means of safely stiffening diseased or surgically weakened corneas. Furthermore, the recent demonstration that significant corneal stiffening can be achieved using a much shorter treatment time than described here (involving only a 5-min NIR exposure)^43^, may further increase its appeal as an alternative to riboflavin/UVA cross-linking.

## Data Availability

The analyzed datasets from this study are available from the corresponding author upon request.
